# Tactile-evoked EEG Dataset for Natural Perception Using an Integrated Stimulation-Recording Framework

**DOI:** 10.1038/s41597-025-06250-8

**Published:** 2025-12-11

**Authors:** Linna Mao, Peishuai Liu, Jingyang Li, Xuhui Wang, Hengjie Su, Xin Zhang, Jing Sun, Ting Li

**Affiliations:** Institute of Biomedical Engineering, Chinese Academy of Medical Sciences & Peking Union Medical College, Tianjin, 300192 China

**Keywords:** Perception, Biomedical engineering

## Abstract

The increasing demand for assistive living and medical technologies in aging societies has driven advancements in tactile-evoked Brian Computer Interface (BCI) systems, offering an alternative to traditional visual and auditory-based BCI systems. However, the development of such systems is constrained by challenges in quantifying tactile sensations and a lack of diverse datasets. This study presents an integrated system enabling natural tactile perception during dynamic touch experience while simultaneously recording electroencephalographic (EEG) responses. EEG signals were collected from 10 healthy participants (64 channels, 1000 Hz) in natural tactile perception tasks involving contact with three distinct materials. Preliminary analysis revealed significant differences in the P300 peak latency and amplitude between tactile conditions, highlighting the unique characteristics of tactile-evoked EEG signals. A three-class classification using Common Spatial Pattern (CSP) and Support Vector Machine (SVM) models demonstrated above-chance accuracy. This tactile-evoked EEG dataset provides a valuable resource for seeking tactile-related neural mechanisms and driving the practical application of BCI systems, offering a pathway to improved user experiences and functionality in real-world scenarios.

## Background & Summary

As the aging of the social population continues to intensify, the growing demand for assistive living and medical devices has propelled research in brain-computer interface (BCI), particularly in health monitoring, assistive instruments, cognitive enhancement, and psychological support^1–4^. Most current Brian Computer Interface (BCI) technologies rely on EEG signals evoked by visual and auditory stimuli^5^, which are susceptible to fatigue and environmental interference^6–8^. In order to serve the requirements of the elderly and individuals with visual or auditory impairments, investigations into tactile-evoked paradigms have further driven the development and application of user-friendly BCI technologies. Compared to visual and auditory modalities, touch is the fundamental sense for humans to explore the external surroundings and engage in direct interaction^9–11^. Tactile exploration is vital for comprehending the physical properties of touched objects and related cognitive processes, providing more information inaccessible via other sensory modalities^12^. However, due to the complexity and difficulty in quantifying tactile perception, theoretical analysis and fundamental research on tactile-evoked EEG signals remain in their infancy and require further exploring.

Numerous studies have been devoted to advancing the tactile-evoked BCI technology, particularly focusing on the neural mechanisms involved in tactile discrimination tasks^13,14^. Most research on tactile BCI has focused on electrical stimulation, with limited attention given to the impact of natural tactile experiences on EEG signals^15–17^. Unlike artificial or electrical stimulation^18,19^, natural tactile stimulation involves physical contact with the external environment, eliciting neurophysiological response through mechanoreceptors in the skin. This gap also restricts the practicality and patient acceptance of the tactile BCI system in real-world applications. Further investigations into the classification of tactile perception, the enhancement of EEG signal processing techniques, and the identification of signals from complex touch experiences contribute to advanced tactile-evoked BCI technology. Recent studies have focused on exploring the neural mechanisms through tactile-evoked EEG signals^20^. Among them, event-related potentials (ERPs), including P300 signals, have been employed to assess tactile sensations and have shown superior performance in the evaluations of various materials^21,22^. The analysis of P300 peak latency and amplitude, evoked by contact with materials of varying roughness, has led to differentiated research findings. While progress is being made in the field of tactile-evoked EEG signals, challenges remain in ensuring the natural authenticity of tactile stimuli and addressing the limited research on the classification of tactile attributes, such as dynamic friction^23,24^. Preserving the complex biomechanical interactions and proprioceptive feedback remains a significant challenge, as further research on neural mechanisms underlying tactile perception continues to be constrained by the limited availability of diverse tactile-evoked EEG signal datasets.

Herein, we developed an integrated stimulation-recording synchronization system for natural, dynamical tactile stimulation and the simultaneous collection of tactile-evoked EEG signals. The real-time perception system setting ensures a natural and realistic touch experience by delivering tactile stimulation through passive touch, in which participants remain still without voluntary movement, thereby enhancing the authenticity and immersion of the perception tasks. We recorded 64-channel EEG signals from 10 healthy participants (all right-handed males, aged 20–26 years) while they performed natural tactile tasks with their left and right hands, involving passive touch with three different types of materials. It is important to note that the participants pool consisted exclusively of healthy, right-handed male adults aged 20–26. While this homogeneous sample helped minimize inter-participant variability and control for potential confounding factors such as sex- or age-related difference, it also limits the generalizability of the findings to broader populations. The high-quality EEG recordings enabled clear discrimination of response to various materials. Analysis of the P300 peak latency and amplitude revealed significant differences in tactile-evoked EEG signals across different materials in several channels. Furthermore, a three-class classification task was performed using a combined Common Spatial Pattern (CSP) and Support Vector Machine (SVM) model, which preliminarily demonstrated the system’s ability to discriminate between materials. This tactile-evoked EEG dataset provides a valuable resource for further investigations into the neural basis underlying tactile perception under naturalistic conditions. It also provides practical support for the development and validation of tactile-based brain-computer interface applications.

## Methods

### Instruments

**Fig. 1:**
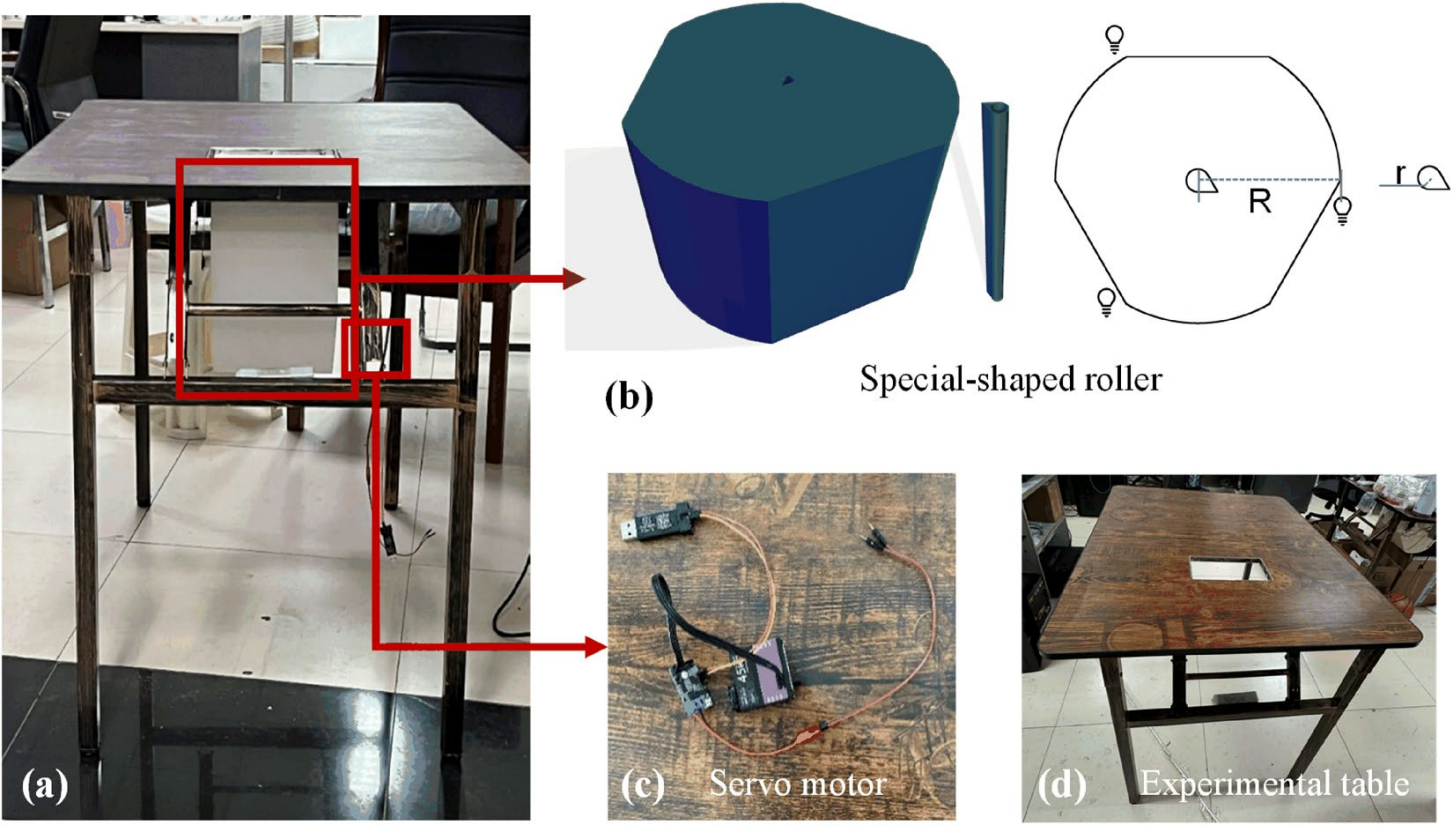
Experimental instruments. (**a**) Experimental setup; (**b**) Special-shaped roller; (**c**) Servo motor; (**d**) Experimental table.

**Fig. 2:**
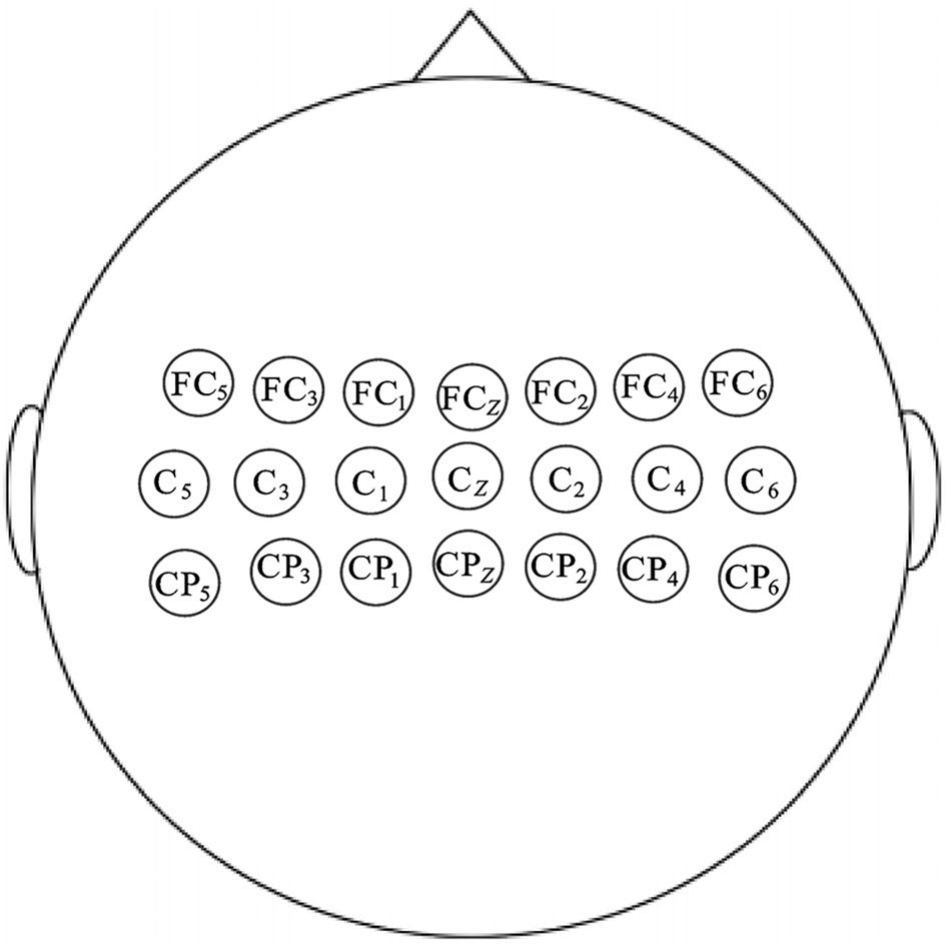
Channel location.

We developed an integrated stimulation-recording synchronization system for implementing a tactile-evoke task, facilitating neuro-EEG data acquisition under various haptic conditions. The designed system comprises two main components: an electromechanical control unit and a hardware assembly consisting of a servo motor, a customized roller, an LED light, and a photoelectric sensor, as shown in Fig. 1. The electromechanical control unit controls the servo motor through host computer software to ensure the accurate execution of tactile stimulation on the participant’s hand. The roller, specially designed for this task, is based on a customized special-shaped roller derived from a regular hexagonal prism, and its three sides are changed from flat to curved, and the flat and curved surfaces are arranged adjacently and alternately. The roller radius is 15 cm, and the height is 20 cm to fit the tabletop slot. The roller’s central axis is waterdrop-shaped to facilitate connection with the linking rod. Three kinds of stimulation materials with obvious differences in tactile sensation were selected from the perspectives of roughness, hardness and friction: artificial leather, artificial fur, and sandpaper^25^. These three simulated materials are respectively attached to the curved surfaces of the special-shaped roller respectively. During the experiment, a cover would be placed on the participants’ hands to prevent visual stimuli from affecting this experiment. An LED light mounted on the side of the roller signals each stimulation event as it passes through the photoelectric sensor. The EEG acquisition device used is the NeuSen W series wireless EEG acquisition device from Bo Ruikang Technology (Changzhou) Co., Ltd., which has 64 channels and a sampling rate of 1000 Hz. Channels FC5, FC4, FC3, FC2, FC1, FCz, C5, C4, C3, C2, C1, CP5, CP4, CP3, CP2, and CP1 were chosen with CPz as the reference, as shown in Fig. 2.

### Procedures

Using a designed integrated stimulation-recording synchronization system to conduct tactile simulation tasks on three different materials. Before the formal experiment, a cognitive familiarization procedure was conducted for each participant. During this session, participants were presented with all stimulus materials and required to correctly recognize and name each material to ensure accurate perception and understanding. At the start of the experiment, the participants are instructed to wear an EEG cap, sit in front of the test bench, and remain resting. Following the instructions, the left and right hands are placed on the test table bench in turn. Throughout the experiment, participants are asked to remain in a sitting state as much as possible. After the experiment begins, the custom-designed roller rotates in a counterclockwise direction. During this process, the three materials attached to the curved surface of the roller stimulate contact and stimulate the palm of the participant in turn. The special-shaped roller is designed with three curved surfaces and three flat surfaces. As the roller rotates, the materials on the curved surface sequentially make contact with the participant’s palm, thereby providing passive tactile stimulation. Contact with the experimental material is recorded as the task state. When it rotates to the flat surface, the participant’s hand is in a suspended state and does not contact the material and is recorded as the resting state. Notably, tactile stimulation during the task state is delivered through natural passive touch, without any active movement or exploration from the participant, thus minimizing subjective influence associated with active behavior. Each full rotation of the roller follows the sequence: artificial fur - rest - artificial leather - rest - sandpaper - rest.

**Fig. 3:**
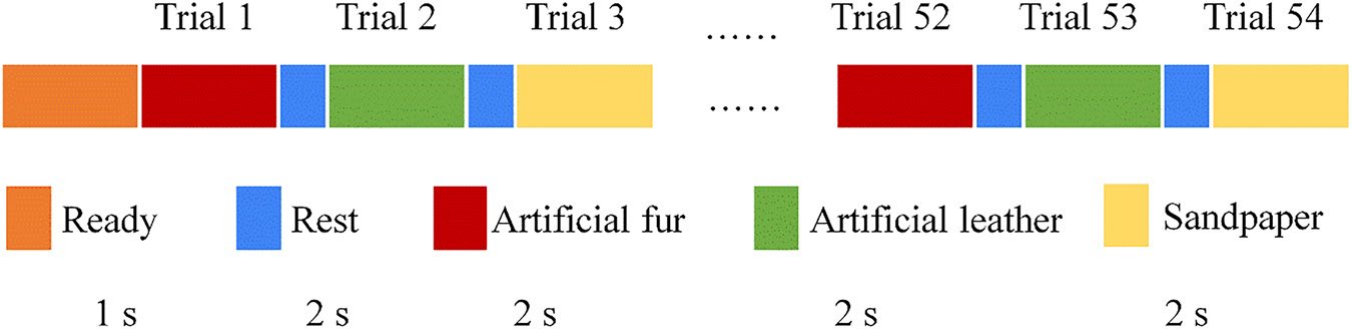
Schematic diagram of experimental protocol.

The experiment consists of two phases: one for the left hand and one for the right hand, each divided into four blocks. After each block, participants rest for 3 minutes. Each block comprises 54 trials, with each trial lasting 4 seconds, including 2 seconds of task engagement and 2 seconds of rest. In each block, the same stimulation is repeated 18 times. After each block, the participants rested for 3 minutes. The detailed experimental process of a single block is shown in Fig. 3.

### Participants

This study was approved by the Ethics Review Committee of Tianjin University (TJUE-2021-019), and 10 healthy participants (ages 20–26 years, male, Right-handed) were recruited. All participants had no history of neurological or musculoskeletal disorders, did not take medications that affect the central nervous system, had no mental health conditions, maintained normal living habits, and had no wounds, scars, or skin conditions on their scalp. All experiments were conducted in accordance with the approved guidelines. All participants were informed and understood the experimental procedures and possible problems before the experiment. After understanding the details and potential risks, all participants provided written informed consent before participating in the study and were fully informed about the nature of the research, including the recording of EEG data. All participants were also informed that the anonymized data collected in this study would be publicly shared for research purposed and provided their consent for such data sharing. Before the experiment, all participants. Participants were required to have a full night’s sleep (8 hours) before the experiment. Naps were prohibited during the experiment, and participants were required to avoid alcohol, tea, nicotine, and caffeine within 24 hours before the experiment.

## Data Record

The open-access dataset we uploaded to Figshare^26^ comprises raw recordings of EEG and has been publicly released at 10.6084/m9.figshare.30479234.v1. Additionally, there is a file named “README.txt” that contains comprehensive information on all open-access data, including data sampling rates, meanings of each parameter, methods for accessing the data, stimulus type sequences, and other pertinent details. The file structure and content of the entire dataset are shown in Table 1.

**Table 1:** Overview of the dataset structure and description.

File/Folder Name	Type	Description
data/mat_doc/	Folder	Contains test data from 10 participants. Each participant is identified by an anoymized code in the format sub_number (e.g., sub_01, sub_02, sub_03, etc.). Each participant’s folder includes multiple ‘.mat’ files named in the format: ‘Hand_block_number.mat’.
data/csv_doc/	Folder	Contains test data from 10 participants. Each participant is identified by an anoymized code in the format sub_number (e.g., sub_01, sub_02, sub_03, etc.). Each participant’s folder includes multiple ‘.csv’ files named in the format: ‘Hand_block_number.csv’. The label file contains the label information corresponding to the csv file.
.mat	Data Files	Each file is an EEG structure compatible with EEGLAB, containing: • EEG.data: EEG signal matrix of shape (channels × time) • EEG.event: event structure with ‘type’ (label) and ‘latency’ (timestamp in samples)
data_pro.mat	MATLAB Script	A preprocessing script. Users input participant name, tested hand, and block number as prompted. The script performs: • Data import • Bandpass filtering (0.1–15 Hz) • Segment extraction from 0–2 seconds after event onset
README.txt	Text File	Provides a description of the included files and instructions for data usage.

## Technical Validation

### EEG data processing

Based on the previous studies, the tactile-evoked brain activity had strong correlations with the kind of fabrics via analyzing the event relative potential P300 signals^27,28^. As an important component of event-related potentials, the P300 ERP is considered to be highly related to the cognitive process of the brain and can be detected during cognitive tasks^29^. The difference in ERP evoked by materials of different roughness was demonstrated using P300 peak latency and amplitude^21^. Therefore, the peak latency and amplitude of the P300 signal for contact stimulation of three different materials were extracted respectively. The P300 amplitudes and peak latencies for all channels for each stimulus were extracted. Then paired t-tests were used for paired data.

The P300 is a critical component of the event-related potentials (ERPs) and typically appears around 300 ms. The filter from 0.1 Hz to 15 Hz was first employed to filter the original data, and then all the EEG signals under the same stimulus on the same participant’s ipsilateral hand were superimposed and averaged^30,31^. The peak potential of each channel between 250 ms and 450 ms was obtained, and the peak closest to 300 ms was considered to be the peak potential of P300, and the time corresponding to this point is considered to be the peak latency of P300. To further explore the influence of stimulus condition and stimulated hand on EEG signals, we used a two-factor repeated-measures ANOVA on the obtained P300 peak latency and amplitude of all channels, with stimulus condition and stimulated hand as factors, to determine which factor significantly influences the participant’s ERP. When the results P < 0.05, the factor was considered there is a significant difference in the signal of this lead. Furthermore, the Degree of freedom (df) reflects the amount of independent information in the data was calculated when performing statistical tests. In addition, the post hoc tests were performed on the leads that showed significant differences. When P < 0.005, it was considered that there was a significant difference between the two sets of data.

### Classification algorithm

**Fig. 4:**
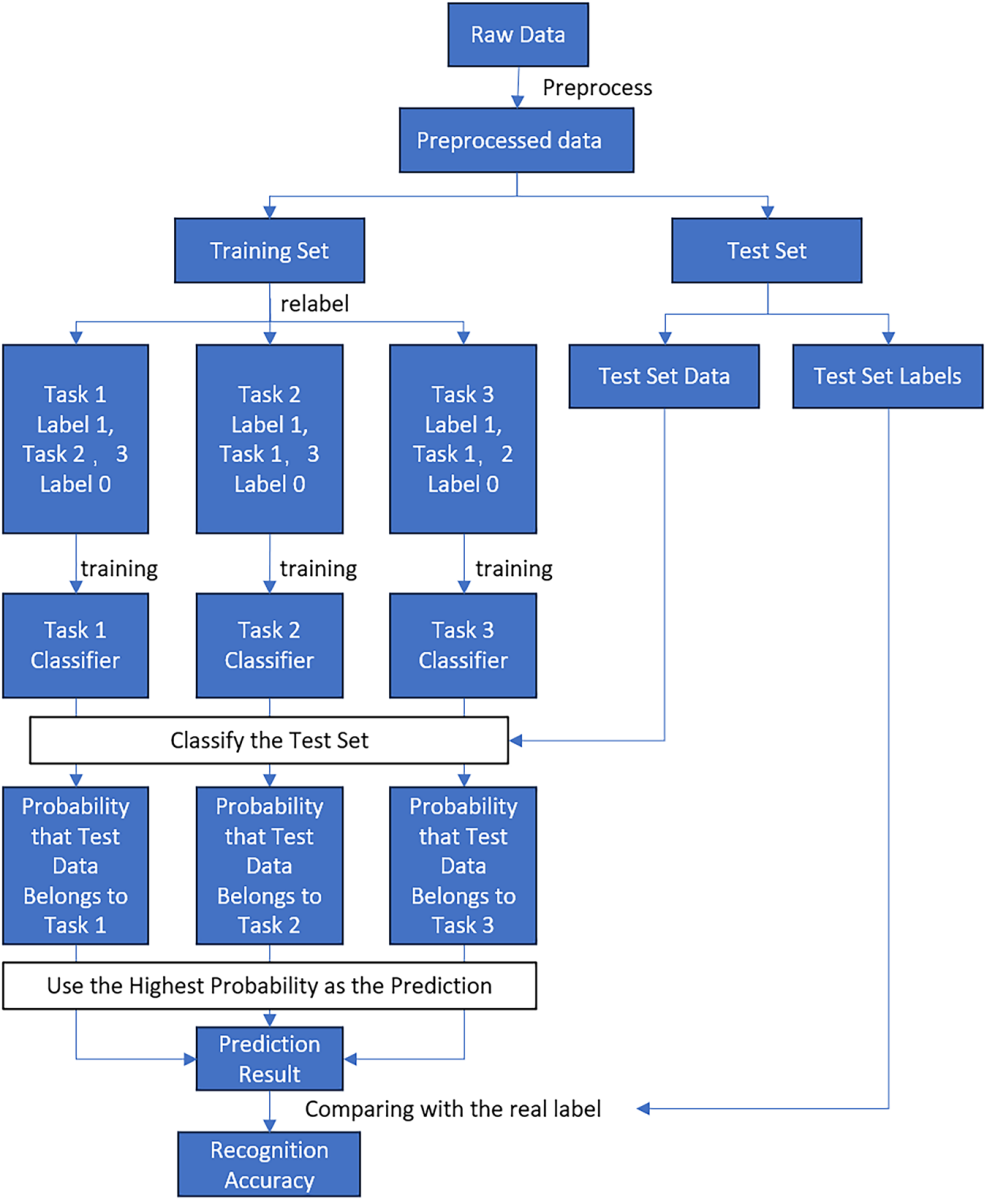
Overview of the EEG signal classification process.

The analysis method of integrating Common Spatial Pattern (CSP) and Support Vector Machine (SVM) is widely used in the classification of EEG signals for its considerable performance^32^. Therefore, the CSP/SVM classification method was employed here to classify the three tactile evoked EEG signals. CSP is used for feature extraction, and then SVM is used for classification. The algorithmic workflow adopted in this analysis is illustrated in Fig. 4.

The common spatial patterns (CSP) algorithm is a feature extraction method that is widely used in EEG feature extraction. The basic principle of CSP is to find a set of spatial filters that effectively differentiate between two classes of signals based on their covariance matrices. The supervised property relies on the accuracy of training sample labels, making it suitable for binary classification tasks. CSP simultaneously diagonalized the covariance matrices of two classes to construct optimal spatial filters, maximizing the difference in energy between the spatial components of the two classes. This is achieved by maximizing the variance of the projected signals of one class while minimizing the variance of the projected signals of the other class^33^. The Support Vector Machine (SVM) is the most widely used machine learning algorithm in classification tasks, with excellent classification ability and computational efficiency. The primary goal of SVM is to find the optimal hyperplane that best separates data points of different classes in a high-dimensional space^34^.

Based on previous studies, both CSP and SVM are oriented to binary classification tasks. In order to apply this method to the three-classification task of this work, a combination of CSP and SVM based on a one-vs-rest strategy was employed. Three CSP/SVM models were developed, each used to differentiate between artificial leather and other materials, artificial fur and other materials, and sandpaper and other materials. The raw data was first preprocessed, and then the aforementioned CSP/SVM models were applied to the processed data. Each CSP/SVM model generates corresponding classification probabilities, which are used to predict the labels of the input data. After the preprocessed data is classified by the three models, the probability of the data group being the three materials will be obtained, and the predicted labels will be gained and therefore obtain the classification accuracy and confusion matrix. We used the SVC function from the scikit-learn library for classification. The specific parameters are as follows: class_weight is set to ‘balanced’, the regularization parameter (C) uses the default value C = 1.0, and the kernel function parameter adopts the default setting ‘scale’. We used a customized implementation of the Common Spatial Pattern (CSP) algorithm for spatial filtering and feature extraction. The specific parameter is the number of spatial filters (m_filters), which was set to 6 in our study. This means that three filters with the highest eigenvalues and three with the lowest were selected, resulting in a total of 6 features per trial. The covariance matrices were normalized by their trace to ensure comparability across trials, and the final features were computed as the logarithm of the variance of the spatially filtered signals.

The whole data was divided into training and validation sets. Each participant contributed 216 samples per hand, with 24 samples (8 for each type of stimulus) designated as the test set, and the remaining 192 samples used for training through an 8-fold cross-validation process. During each fold, 168 samples (56 for each type of stimulus) were used to train the classifier, and 24 samples (8 for each type of stimulus) were used to validate the classifier. After completing the 8-fold cross-validation, the classifier with the highest recognition accuracy was selected to classify the data in the test set, thereby obtaining the recognition accuracy for each participant. To investigate whether the stimulus condition and the hand being stimulated affect classification accuracy, paired t-tests were conducted on the classification results. A significant difference was considered present when P < 0.05. Furthermore, to explore the influence of different hands and stimuli on recognition accuracy, a two-factor repeated-measures analysis of variance (ANOVA) was performed on the recognition accuracy of the three types of stimuli derived from the confusion matrix, with stimulus condition and stimulated hand as factors.

### EEG results

The peak latency of the P300 component is generally interpreted as the speed of stimulus classification resulting from distinguishing one event from another, making this parameter a classification speed indicator proportional to stimulus evaluation time. This parameter also varies with individual differences in cognitive ability. Results from a two-factor repeated-measures analysis of variance (ANOVA) across all channels indicate that stimulus type has a significant effect on the P300 peak latency at the Cz channel (P = 0.012, df = 2, 18) and the CP2 channel (P = 0.0338, df = 2, 18). Additionally, there is a significant interaction effect of hand stimulated and stimulus type at the CP5 channel (P = 0.04, df = 2, 54) and CP6 channel (P = 0.011, df = 2, 54). P300 peak latency data for channels Cz, CP2, CP5, and CP6 are presented in Table 2.

**Table 2:** P300 peak latencies of channel Cz, CP2, CP5, and CP6.

Channel	Left hand			Right hand		
	AL	AF	SP	AL	AF	SP
Cz	−29.1 ± 29.767	2.3 ± 34.648	16.2 ± 16.315	−0.2 ± 26.394	10.7 ± 26.621	7.8 ± 56.543
CP2	−13.1 ± 32.402	−0.5 ± 36.834	10.2 ± 32.69	3.6 ± 32.844	−8.6 ± 25.859	32.1 ± 23.038
CP5	−7.3 ± 30.948	−6.6 ± 35.569	16.1 ± 49.314	−10.6 ± 27.714	26.3 ± 42.269	−7.7 ± 38.358
CP6	−17.1 ± 31.232	10 ± 23.514	6.9 ± 25.053	5.6 ± 25.937	−12 ± 20.95	14.2 ± 23.911

(AL: artificial leather; AF: artificial fur; SP: sandpaper).

Besides, post hoc tests were conducted to explore the effects of different stimulus types on P300 peak latency. Specifically, the Bonferroni correction method was applied for pairwise comparisons of the P300 peak latency at Cz, CP2, CP5, and CP6 channels. The results indicated a significant difference in P300 peak latency at the Cz channel between artificial leather and sandpaper stimuli for left-hand stimulation (P = 0.039, df = 9). At the CP2 channel, a significant difference in P300 peak latency was observed between artificial leather and sandpaper stimuli (P = 0.031, df = 9). Additionally, for the right hand, a significant difference was found between artificial fur and sandpaper stimuli (P = 0.011, df = 9).

Meanwhile, P300 peak amplitudes were also analyzed and the results indicated that stimulus type had a significant effect on the P300 amplitude at the Cz channel (P = 0.036, df = 2, 18). At the C1 channel, there was a significant interaction effect between stimulus type and stimulated hand (P = 0.004, df = 2, 18). P300 peak amplitudes for the Cz and C1 channels are presented in Table 3.

**Table 3:** P300 peak of channel Cz and C1.

Channel	Left hand			Right hand		
	AL	AF	SP	AL	AF	SP
C1	31.3 ± 16.028	22.8 ± 18.861	18.2 ± 13.307	27.7 ± 15.166	19.9 ± 19.485	49.8 ± 25.183
Cz	37.7 ± 15.741	18.6 ± 13.167	23.7 ± 14.553	30.1 ± 14.13	20.8 ± 14.695	43.2 ± 34.512

(AL: artificial leather; AF: artificial fur; SP: sandpaper).

### Classification results

**Fig. 5:**
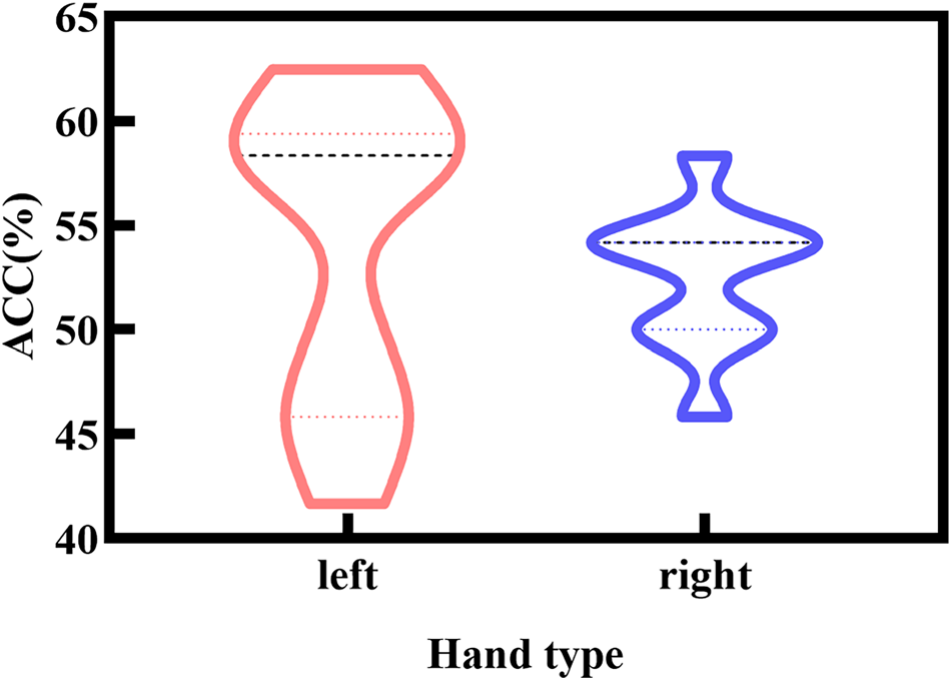
Violin plots showing the distribution of three-class classification accuracies for the left and right.

This study conducted a three-class classification of EEG signals evoked by three types of tactile stimuli—artificial leather, artificial fur, and sandpaper—on the left and right hands. As shown in Fig. 5, the results indicated that the classification accuracy for the left and right hand was 54.167 ± 7.607% and 52.5 ± 3.514%, respectively. The results of a paired t-test showed no significant differences between the two hands.

**Fig. 6:**
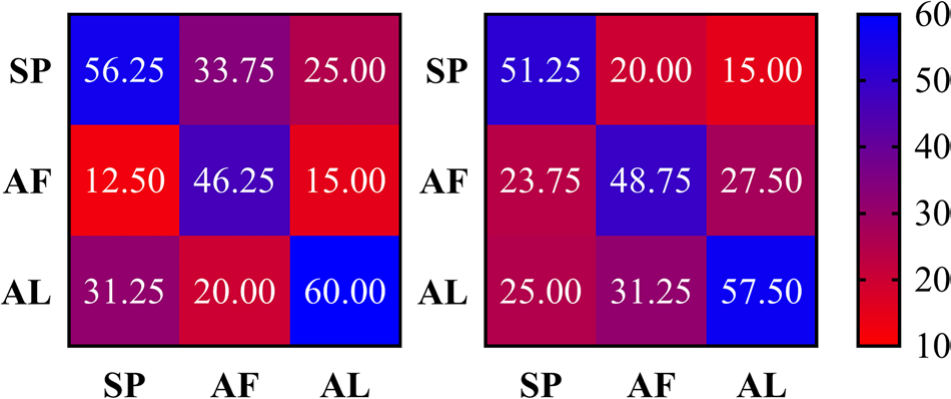
Three-class confusion matrix results. (AL: artificial leather; AF: artificial fur; SP: sandpaper).

To further investigate this, we plotted the confusion matrix for all participants’ averaged data for the left and right hands, as shown in Fig. 6. From the confusion matrix, it is evident that the recognition accuracy for sandpaper stimulation was the highest for both the left and right hands, followed by artificial leather stimulation, and finally, artificial fur stimulation. Additionally, this result may be attributed to the fact that all participants in this study were right-handed.

## Usage Notes

This dataset includes EEG signals from 10 healthy participants (10 males, aged 23.0 ± 2.3 years, all right-handed) during a tactile evoked potential experiment, with 21 channels recorded at a sampling frequency of 1000 Hz. These data can be used to explore the patterns of EEG signals evoked by different materials, as well as for constructing a brain-machine interface system based on tactile evoked potentials.

From the perspective of exploring the patterns of tactile evoked EEG signals, significant results were observed in the P300 peak latency or amplitude at the Cz, CP2, CP5, CP6, and C1 channels. From the perspective of constructing a brain-machine interface system based on tactile evoked potentials, the recognition accuracy of the model in the early three-class classification task was greater than 50%. Considering the complexity of the data and the challenges of multi-class classification tasks, this accuracy still provides valuable preliminary insights.

## Data Availability

The dataset is available in the Figshare Repository at the following URL: 10.6084/m9.figshare.30479234.v1. All shared data have been fully anonymized, and participants provided informed content for their public release.

## Code availability

The data processing program is recommended to be opened using MATLAB 2023. In processing EEG data, the MATLAB plugin EEGLAB should be used for preprocessing the data of each participant. Detailed instructions can be found in the official EEGLAB user manual. We also provide reference data processing scripts, which are available on GitHub at https://github.com/liupeishuai/Code-for-Tactile-evoked-EEG-Dataset. No custom code was used in this study.
